# Telemedicine in the post-COVID era: balancing accessibility, equity, and sustainability in primary healthcare

**DOI:** 10.3389/fdgth.2024.1432871

**Published:** 2024-08-21

**Authors:** Waseem Jerjes, Daniel Harding

**Affiliations:** ^1^ Research and Development Unit, Hammersmith and Fulham Primary Care Network, London, United Kingdom; ^2^ Faculty of Medicine, Imperial College London, London, United Kingdom

**Keywords:** telemedicine, accessibility, equity, sustainability, primary healthcare

## Introduction

In the wake of the COVID-19 pandemic telemedicine has rapidly ascended as a pivotal component of healthcare delivery (1). The unprecedented global health crisis catalysed a surge in the adoption of digital health technologies, with telemedicine at the forefront (2). This transition not only highlighted the versatility and potential of telemedicine to support healthcare systems but also exposed a range of challenges and disparities inherent in its widespread implementation. The surge in telemedicine usage highlighted critical issues around accessibility, particularly for marginalised groups, underscoring the urgent need to address these disparities to harness the full potential of telehealth (3).

Telemedicine has allowed unprecedented accessibility for those with mobility or geographical restrictions while also enabling the continuation of care when social distancing is required (4). However, it simultaneously exposes issues related to the digital divide healthcare equity and the long-term sustainability of such services (5). There is a critical need to address these challenges to ensure that the benefits of telemedicine can be universally accessed and sustained (6). Research indicates that while telemedicine can mitigate transportation and time-related barriers it may also exacerbate existing disparities in digital access and literacy (7, 8).

To provide context this opinion paper focuses on the primary care setting within the NHS framework in the UK where unique challenges and opportunities for telemedicine have emerged. Furthermore, the objective of this opinion paper is to critically analyse the balance between the potential benefits and the multifaceted challenges presented by telemedicine.

By exploring these dimensions, we aim to propose integrated solutions that enhance the role of telemedicine in primary care. This analysis incorporates recent findings on telemedicine's impact on healthcare accessibility equity and sustainability with a focus on strategies to overcome identified barriers.

### Digital divide and accessibility challenges

The term “digital divide” refers to the gap between individuals who have access to modern information and communication technology, and those who do not (9–11). This divide encompasses a spectrum of factors, including geographical, socioeconomic, and demographic elements, which collectively influence the efficacy of telemedicine initiatives (1, 12). In this context the digital divide represents a significant hurdle in the quest for equitable healthcare provision. Recent studies have underscored that digital literacy and access to technology are pivotal in determining telemedicine's success in bridging healthcare gaps (13).

Geographical isolation remains a formidable barrier in many parts of the UK particularly in rural and remote areas where broadband infrastructure is inadequate (1, 14). The implications are profound: without reliable internet access, residents in these areas are often excluded from the telemedicine revolution, thus perpetuating existing healthcare disparities (15). This challenge contrasts with the previously mentioned accessibility benefits highlighting the complex and dual nature of telemedicine's impact on different regions (16). Efforts to improve digital infrastructure in rural areas have shown promise but require sustained investment and policy support (17).

Moreover, socioeconomic status plays a crucial role in determining an individual's ability to engage with telemedicine (18). The costs associated with digital access (broadband service or mobile data, digital devices, and ongoing maintenance) can be prohibitively expensive for those experiencing deprivation (19). Consequently, even in urban areas where technological infrastructure is not a challenge economic barrier can still restrict access to telemedicine services (20). Addressing these economic barriers is essential for equitable telehealth implementation as highlighted in various studies emphasising the need for subsidies and affordable technology solutions (21).

The interplay between age and digital literacy also significantly affects telemedicine's accessibility (22). Although older populations often exhibit lower levels of digital literacy this stereotype is evolving. Many older adults are increasingly motivated to learn and use digital health tools especially when it enables them to receive care at home. Thus, considerations for digital literacy need to address all age groups (2, 23). Innovative educational programs targeting digital literacy across demographics have been effective in enhancing engagement with telehealth services (24).

Addressing these challenges necessitates a multifaceted approach (25). It is imperative that healthcare policymakers and stakeholders recognise the breadth of the digital divide and implement strategies that are inclusive of all societal segments (Table 1) (26). This includes investing in digital infrastructure to ensure widespread reliable internet access subsidising the cost of digital technology for low-income families and developing targeted educational programs that increase digital literacy across all age groups (2, 27). Successful case studies have demonstrated that community-based interventions such as telemedicine booths in public spaces can significantly enhance access and usability for underserved populations (28).

**Table 1:** Challenges, impacts, and strategic solutions in telemedicine.

Challenge	Impact	Strategic solution	Section
Geographical barriers	Limits access in rural and remote areas	Invest in expanding digital infrastructure	Access
Socio-economic disparities	Economic barriers restrict access for low-income households	Subsidise technology costs; negotiate affordable rates	Equity
Digital literacy	Affects elderly and socio-economically disadvantaged	Develop targeted educational programs in community centres	Access, Equity
Language and cultural barriers	Creates access issues for non-English speakers and diverse cultures	Integrate translation services and cultural competence training	Equity
Physical and sensory disabilities	Standard platforms may not accommodate all users	Adapt platforms with accessible features (e.g., high-contrast visuals, voice-to-text functionality)	Equity
Legal and ethical issues	Necessity to protect patient privacy and ensure equitable access	Establish clear legal frameworks and ethical guidelines	Equity
Sustainability	Financial and infrastructural challenges impacting long-term viability	Public-private partnerships, governmental subsidies, and sustainable funding models	Sustainability
Continuity of care	Challenges in maintaining consistent care across different healthcare settings	Promote interoperability among health systems; standardise telemedicine practices	Sustainability

### Equity concerns in telemedicine

In the rapidly evolving landscape of telemedicine equity concerns have become increasingly salient (29). As we harness digital platforms to enhance healthcare delivery it is imperative to scrutinise equity within these technological advances to prevent it paradoxically and inadvertently perpetuating existing inequalities (30). The design and implementation of telemedicine systems must prioritise equity to ensure that they do not exacerbate existing health disparities (31).

Central to the discourse on equity within telemedicine is the challenge of language barriers and cultural competence (32). Patients from non-English speaking backgrounds often encounter difficulties in accessing telemedicine services that are predominantly designed for English-speaking users (33). This not only impedes their ability to receive care but also affects the quality of the healthcare delivered as misunderstandings and miscommunications are more likely to occur risking disparities in health outcomes (34). Integrating robust language support and cultural sensitivity into telehealth platforms has been shown to significantly improve access and patient satisfaction among diverse populations (35). Additionally speech-to-text functionality and scalable text options can significantly improve usability for individuals with sensory impairments ensuring that language barriers and disabilities do not impede access to healthcare (36).

The design of telemedicine services must also include considerations for individuals with cognitive impairments or mental health conditions (37). Features like memory aids, simple decision-making tools, and interfaces that consider neurodiversity needs can make telemedicine a viable option for those who might find traditional health service settings overwhelming (1, 8). This is a major benefit of telemedicine providing more personalised and accessible healthcare options that cater to specific patient needs (22, 29).

To address these equity concerns it is essential that telemedicine providers and policymakers take active steps toward inclusivity and adaptability in the design and deployment of digital health services (3, 30). This might include the integration of multilingual support and cultural sensitivity training (32, 33). Additionally telemedicine platforms should be developed and tested with input from diverse user groups to ensure they are accessible and user-friendly for all (5). Collaborative efforts between public health agencies and community organisations are crucial in developing telehealth solutions that truly address the needs of all population segments (18, 22). This commitment to inclusivity can not only improve individual patient outcomes but also contribute to the broader objectives of equity and sustainability in the healthcare system (7).

### Sustainability of telemedicine

For telemedicine to be sustainable as a primary healthcare modality, it must address several critical financial and infrastructural challenges (8, 30, 36). Constant technology updates, cybersecurity needs, and continuous training of healthcare professionals are major cost drivers in maintaining telemedicine services (15, 19). These costs can be particularly burdensome for health systems already under fiscal pressure (6, 9, 32). Without sustainable funding models, the initial enthusiasm for telemedicine might wane, leading to a decline in quality and service (11). Therefore, innovative funding solutions, such as public-private partnerships and governmental subsidies, are essential to support the long-term viability of telemedicine (12, 29).

Successful telemedicine infrastructure requires not only hardware but also integrated electronic health records from various health and social care providers, data management systems, and secure communication platforms (13). High levels of data integration and interoperability are crucial for optimising telehealth services and ensuring sustainability (14). Utilising the most advanced and accurate digital technologies is key to maintaining sustainable telehealth services.

Governments play a crucial role in legislating the adoption of telemedicine (9, 22). They can significantly influence the sustainability of telemedicine by funding its infrastructure through strategic investments, especially in rural or underserved areas where the digital divide is most pronounced (16). This includes enacting laws to ensure patient privacy and clear guidelines for telemedicine practice (1–3). Policy frameworks supporting sustainable telehealth practices are essential for operationalising telemedicine from conceptualisation to practical utilisation (19).

The private sector's involvement is also vital for advancing telemedicine by driving innovations that make it more accessible, user-friendly, and efficient (20, 33). However, it is crucial to ensure telemedicine remains affordable and free from monopolistic practices (2, 6, 19). Collaborations with the private sector can lead to significant advancements in telehealth technology, provided that affordability and accessibility are prioritised (22, 37).

Policy formulation should address financial and infrastructural improvements to enhance sustainability while integrating telemedicine services into a general health service planning framework (23). Policies should also promote interoperability among health systems, standardise telemedicine practices to ensure quality and safety, and encourage data sharing under strict privacy standards (1, 2, 4). Including telemedicine in mainstream healthcare settings offers an opportunity to improve coordination and efficiency in healthcare delivery (27).

### Integrating telemedicine with traditional healthcare

In its entirety, the integration of telemedicine with traditional health care is a critical factor in the transformation of health services delivery processes today (1–3). Since primary health care encompasses an exceedingly dynamic field, telemedicine ought to be envisioned not as a replacement for in-person health care but rather as an extension and enhancement of in-person health care (27). This should include standardised criteria and guidelines for the selection of telemedicine platforms, vetting platforms, and best practices on how to use the technology and the clinical workflows (28). In addition, more education investment is needed with new models of delivery care, mostly for the younger generation of health providers who will drive innovations in telehealth (29).

Robust protocols are essential to delineate when and how telemedicine should be integrated with traditional healthcare practices (6, 18). These protocols should explicitly define the situations where telemedicine is the most appropriate mode of care and when in-person consultations are required (31). For instance, while telemedicine is suitable for managing chronic conditions or follow-up visits, it is less appropriate for initial diagnoses of complex conditions that necessitate physical examination.

Furthermore, the process of integration has to take into account the operational matters concerning healthcare provision (36, 37). This encompasses scheduling of appointments, managing patient records, interservice referrals, and coordination of care across platforms and providers (34). Proper logistics management should be put in place for continuity of care regardless of the modality used (1–3).

To achieve these goals, continuing education and the support of health care providers is critical (4, 19). Such training should encompass both technical aspects in handling telemedicine technologies and clinical skills to provide quality care remotely—besides realising telemedicine limitations and being able to discriminate about when to shift to in-person care (1).

Ultimately, the integration of telemedicine into traditional healthcare settings should be guided by the principle of patient-centred care (6, 18). This means placing the needs and preferences of patients first, making sure they are well informed in all their care options, and being involved in the decision-making process. Through a collaborative approach that respects and addresses the patient's condition, health care providers are able to use the strengths of both telemedicine and traditional care modalities (3, 19). This also falls under the wider heading of equity in that equitable access and patient involvement are key to effective incorporation of telehealth.

### Legal and ethical considerations

The COVID-19 pandemic has created a surge in telemedicine practices across the world, complementing the urgent need for strong legal frameworks and ethical guidelines (4, 19). Legal considerations, on the other hand, are particularly focused on the protection of patient privacy, a fundamental right that becomes vulnerable in digital interactions (8). Lastly, the considerations of an ethical nature revolve around the equitable provision of services (7).

The legal landscapes for telemedicine are fraught with complexities, including matters on data security and cross-border regulation of telehealth services, both of which are rather complex (6, 12, 20). Patient data could be potentially breached in the digital sphere, leading to an infringement on one's personal privacy or sensitive health information being misused (9). That is why the services have to comply with very strict regulations in terms of data protection (22). This is fundamentally no different from the risks associated with electronic health records, hence underlining the necessity of consistent and rigorous security protocols across all digital health platforms (11).

Besides this, telemedicine has created a jurisdictional ambiguity because one provider and the other party might be in different regions, hence the question of which kind of legal frameworks control this kind of interaction (19, 24). The law must also be clear that the standards of care set are no different from those of an in-person consultation irrespective of the location of the patient or the provider (14, 28).

The expansion of telemedicine raises serious ethical problems regarding access and equity (1–3); the so-called digital divide represents both an ethical and technological problem (14, 16). Efforts need to be made to bring telemedicine to all sectors of the people, which could be achieved by subsidising or making inexpensive the internet and technological development in underprivileged areas and among populations (18). If anything, telemedicine should add to the ability to serve and not create a parallel system that would segregate care into digital and non-digital categories. Policy and practice should secure that all patients, irrespective of their socioeconomic status, age, ability, or geographic location, have equal access to all forms of care.

Patient consent is another cornerstone of ethical telemedicine practice (6, 9, 30). It therefore ought to understand fully what the practice of telemedicine entails and the scope of data to be shared. In telemedicine, informed consent may be challenging but necessary; thus, clear communication strategies may have to be accompanied by new approaches that are amenable to digital comprehension.

## Discussion

In placing the many dimensions of telemedicine in perspective, especially following the COVID-19 pandemic, it is critical to synthesise our findings with a view toward proposing some real solutions that accommodate the dual objectives of expanded accessibility and sustained equity (24). The discussion regarding telemedicine, particularly in the context of primary health care, similarly requires a comprehensive consideration that assures the level at which technological innovation is deployed within the health care system is based on patient-centred traditional practices, where digital health tools serve to augment this process rather than as a stand-in, either wholly or partially, for these traditional medical interactions (25). For example, evidence suggests that community-based telemedicine programs have worked and increased access in rural areas, whereas public-private partnerships have demonstrated success in bringing low-cost telehealth services to underserved populations.

The synthesis of this range of challenges underscores the critical importance of a strategic framework that takes into account not only the technological and infrastructural requirements but also their ethical and legal dimensions in regard to telemedicine (9, 19, 20). Such a framework shall aim at strengthening the resilience and capability of health systems to deliver continuous comprehensive and equitable care across diverse populations (28). Frameworks aimed at addressing equity in telemedicine must take into account the special needs of the different populations of patients, and any telehealth solution arrived at should be comprehensive and flexible (29).

Additionally, as we chart the future for telemedicine, this cannot escape innovation and research in the same field (35, 36). Constant progress in technology and iterative feedback from providers and patients are crucial for continuously tailoring and honing telemedicine tools and practices, not only for the present demands but also for future needs (1, 8). It is important that engagement with academic institutions, technology experts, and community organisations develops a rich knowledge and innovation ecosystem in telemedicine (19). The critical areas for collaboration are, therefore, those important for the generation of insights into best practices and innovative solutions, which maximise ability and equity within telemedicine (2, 19).

We further argue that strong community engagement and policy advocacy are the most basic components that drive telemedicine to higher levels (35). If awareness on benefits, equity, and challenges in access of telemedicine services can be made, health care providers can be at the forefront in making policies that can guide responsible integration of digital tools into standard health care practices. Through community-based interventions and public health campaigns, the acceptance and utilisation of telemedicine services increase significantly (Table 2).

**Table 2:** Solutions from other settings and their applicability to primary care.

Challenge	Solution in other settings	Viability in primary care (UK)	Remaining gaps and considerations
Digital literacy	Digital literacy workshops at community centres	Highly viable; can be implemented through NHS community programs	Need for tailored programs for various demographics; continuous updates required
Broadband access	Rural broadband initiatives and subsidies	Moderately viable; requires significant investment	Persistent connectivity issues in extremely remote areas
Language barriers	Multilingual telehealth platforms	Highly viable; necessary in diverse urban areas	Limited availability of trained multilingual staff
Cost of technology	Free or low-cost devices for low-income households	Viable with governmental and private sector collaboration	Ensuring long-term sustainability and device maintenance
Data security	Advanced encryption and regular audits	Highly viable; aligns with NHS data security standards	Need for regular updates and compliance monitoring
Integration with EHR	Unified electronic health record systems	Viable; enhances coordination across NHS trusts	Standardisation and compatibility issues across different systems
Patient engagement	User-friendly telehealth applications	Highly viable; improves patient involvement and satisfaction	Ensuring accessibility for elderly and disabled patients
Cultural competence	Training healthcare providers in cultural competence	Highly viable; enhances patient-provider communication	Continuous training and assessment needed
Sustainable funding	Public-private partnerships and grants	Viable; stabilises funding for telehealth programs	Dependence on ongoing financial support; economic fluctuations
Remote monitoring	Use of wearables and IoT devices	Moderately viable; enhances chronic disease management	High initial costs and patient adherence to using devices

Finally, a call to action for primary care physicians and healthcare providers is imperative. They will be the frontline agents in the integration of telemedicine—not simply in its adoption but, more critically, in being leaders of practice that advocate and implement systems to ensure that telemedicine is sustained, fair, and effective. This workforce should be properly equipped with the tools and knowledge to navigate the constantly evolving telehealth landscape and to advocate for patient-centred solutions in digital health. Primary care providers, among these professionals, should take part in continuous learning, patient education, and policy advocacy to embrace the full potentials of telemedicine—otherwise, it will pose a risk of becoming a less effective or equitable parallel to in-person care.

In conclusion, the obstacles of telemedicine are therefore huge but they must not be insurmountable. Technological investment, policy overhauls, and active engagement in taking care of healthcare professionals and patients will enable telemedicine to transcend its current limitations into a mainstay of modern, equitable, and resilient healthcare. The path ahead is one of collaboration, innovation, and persistent advocacy in which the potential benefits to patient care and system efficiency are rich.
